# Multifragmentary patellar fracture has a distinct fracture pattern which makes coronal split, inferior pole, or satellite fragments

**DOI:** 10.1038/s41598-021-02215-0

**Published:** 2021-11-24

**Authors:** Jae-Woo Cho, Zepa Yang, Eic Ju Lim, Seungyeob Sakong, Wonseok Choi, Whee Sung Son, Hanju Kim, An Seong Chang, Do-Young Lim, Youngwoo Kim, Beom-Soo Kim, Jong-Keon Oh

**Affiliations:** 1 Department of Orthopedic Surgery, Korea University Guro Hospital, College of Medicine, Korea University, 148, Gurodong-ro, Guro-gu, Seoul, 08308 Republic of Korea; 2 Department of Radiology, Korea University Guro Hospital, College of Medicine, Korea University, Seoul, Republic of Korea; 3 Department of Orthopaedic Surgery, Chungbuk National University Hospital, Chungbuk National University College of Medicine, Cheongju, Republic of Korea; 4 Department of Orthopedic Surgery, Uijeongbu St. Mary’s Hospital, College of Medicine, The Catholic University of Korea, Uijeongbu, Republic of Korea; 5 Department of Orthopedic Surgery, Dongsan Medical Center, School of Medicine, Keimyung University Daegu, Daegu, Republic of Korea; 6 Department of Orthopaedic Surgery, Keimyung University School of Medicine, 1035, Dalgubeol-daero, Dalseo-gu, Daegu, 42601 Republic of Korea

**Keywords:** Trauma, Translational research

## Abstract

The present study aimed to map the location and frequency of fracture lines on the coronal articular and sagittal planes in multifragmentary patellar fractures. 66 multifragmentary patellar fractures were digitally reconstructed using the 3D CT mapping technique. The coronal articular surface and midsagittal fracture maps were produced by superimposing each case over a single template. Each fracture line was classified based on the initial displacement and orientation. We evaluated the frequency and direction of the fracture line, coronal split fragment area, and satellite and inferior pole fragment presence. Coronal articular surface fracture mapping identified primary horizontal fracture lines between the middle and inferior one-third of the articular surface in 63 patients (95.4%). Secondary horizontal fracture lines running on the inferior border of the articular facet were confirmed (83.3%). Secondary vertical fracture lines creating satellite fragments were mostly located on the periphery of the bilateral facet. Midsagittal fracture mapping of primary and secondary horizontal fracture lines with the main coronal fracture line revealed a predominantly X-shaped fracture map. The consequent coronal split fragment and inferior pole fracture were combined in most cases. In conclusion, the multifragmentary patellar fracture has a distinct pattern which makes coronal split, inferior pole, or satellite fragments.

## Introduction

Although patellar fractures represent only 0.5–1.5% of all skeletal injuries^1,2^, they can be significant, often resulting in poor functional outcomes if not properly treated. Since these injuries potentially compromise the extensor mechanism, impairment of patellofemoral articulation can directly affect the ability to perform daily activities and the quality of life^3–6^. Therefore, when treating a patellar fracture, it is important to achieve, as accurately as possible, an anatomic reduction of the articular surface in addition to restore the extensor mechanism.

Patellar fractures requiring surgery are comminuted (multifragmentary) type fractures in up to 55% of the cases^1,7^. A recent computed tomography (CT) study revealed that distal pole patellar comminution was noted in 88% of the cases, indicating a substantial prevalence of comminution among fracture cases^8^. Furthermore, comminuted patellar fractures were frequently associated with coronal split fractures and inferior pole comminution^8–11^. This complex fracture type, which is described as a complete articular frontal/coronal multifragmentary patellar fracture, is classified as a 34C3 fracture, according to the current AO/OTA classification^12,13^. This classification system is based on the anterior/posterior view of the patellar fracture without any subgroup categorization, leading to a limited explanation of complex articular fracture patterns.

Detailed knowledge of common fracture patterns and of locations of articular impaction or free articular fragments would aid in simplifying the sequence of fracture reduction and selection of the optimal fixation methods in patellar fracture management. However, limited literature is available regarding anatomic patterns of articular injuries in comminuted patellar fractures, thereby, necessitating further evaluation. The present study aimed to define the location and frequency of fracture lines on the coronal and sagittal planes of the articular surface and then to map the locations of articular impaction in AO/OTA 34C3 fractures.

## Materials and methods

### Subjects

This retrospective study included 142 patellar fractures that were surgically treated at a single university hospital between January 2015 and December 2018 and approved by our institutional review board (NO:2019GR0364). The individual informed consent for clinical data was obtained for study purposes in outpatient clinic visits. We obtained the CT scans and plain radiographs of each patient’s knee injury. Preoperative CT was performed in 1-mm-thick intervals. The medical records were retrieved to identify the patient demographic characteristics, injury mechanisms, and associated injuries around the knee. The present study excluded patients aged < 18 years and > 71 years and those with a previous fracture or dislocation history of the patella or bipartite patella. As low bone mineral density can dissuade the precise 3D modeling and deteriorate analysis, the patients over 70 from the initial cohort were excluded. Of the 142 patellar fractures recorded, we identified 66 multifragmentary patellar fractures in 66 patients. Using the AO/OTA classification, we classified the multifragmentary patellar fractures as type 34C3 fractures.

### Coronal articular surface fracture mapping

**Figure 1:**
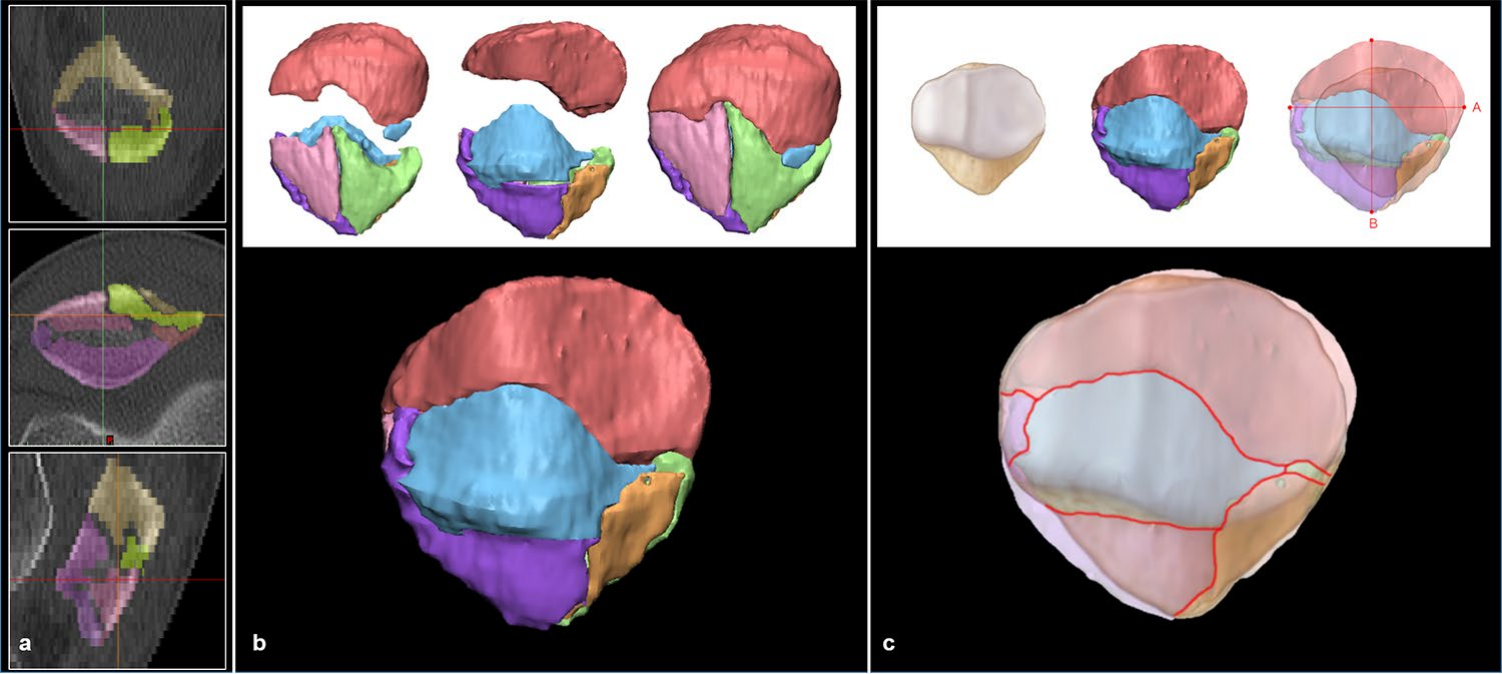
The software employing the method of mapping three-dimensional reassembled fracture models was used for coronal articular surface fracture mapping (**a**). Segmented fracture fragments were reassembled (**b**). Fracture lines that were present on the articular surface (red lines) were drawn on a standard template by superimposing captured images (**c**).

All Digital Imaging and Communications in Medicine (DICOM) files of the preoperative CT scans were imported into Mimics^®^ software (Materialise NV, Belgium) to create a 3D model of each fracture. In the 3D patella model, fractures with over 1 mm displacement were segmented independently. After completing fracture segmentation, the 3D-modeled stereolithography file was imported into 3-matic^®^ software (Materialise NV, Belgium) to reassemble fracture fragments. Each fragment was moved three-dimensionally to fit between nearby fragments. We used fracture margins, the patella’s outer surface continuity, and the shape of the contralateral patella on radiographs as references for fracture reduction (see Supplementary video 1, which demonstrates the Fracture fragment reduction using 3D software). Images of the reassembled articular surface were captured and retrieved in Adobe Photoshop software (Adobe, CA, USA). To superimpose the fracture pattern onto the standard template, we rotated each captured image along the axis of the patellar vertical ridge. Next, we resized the entire articular surface according to facet locations to maintain the image proportions. We then drew the fracture lines in each image on a superimposed template using the pencil tool in Photoshop (Fig. 1). The coronal articular fracture line drawings were created for all 66 cases and merged together to create a coronal articular fracture map.

### Denomination and classification of the fracture lines in coronal articular fracture map

Each fracture line was classified as “primary” or “secondary” and “horizontal” or “vertical” based on the initial displacement and orientation, respectively. The “primary” fracture line was defined as “the largest displaced fracture line on the preoperative 3D CT scans considering both fracture gap or step” and the “secondary” fracture lines as “other displaced fracture lines on the preoperative 3D CT scans.” Fracture lines oriented at an angle < 45° were classified as “horizontal”, while those oriented at a greater angle were classified as “vertical”.

We used the “Fracture line denominating and mapping program” to classify each fracture line and to determine the average value of the classified fracture lines. In this program, each fracture line was sectioned from its start point to its exit point or point of intersection with another fracture line. The sectioned lines were designated their respective categories according to the independent observers’ judgement. The result of each classification was saved as a categorical digital value to make reliability analysis possible.

Two independent observers, trained through an orthopedic trauma fellowship, were involved in the classification process. They denominated the fracture lines in 66 cases individually, according to our working definition of the classification, and repeated the same classification twice with an interval of 14 days. The final denomination of each fracture line was confirmed by reviewing the results of the denominated fracture map by arriving at a consensus with two senior surgeons.

**Figure 2:**
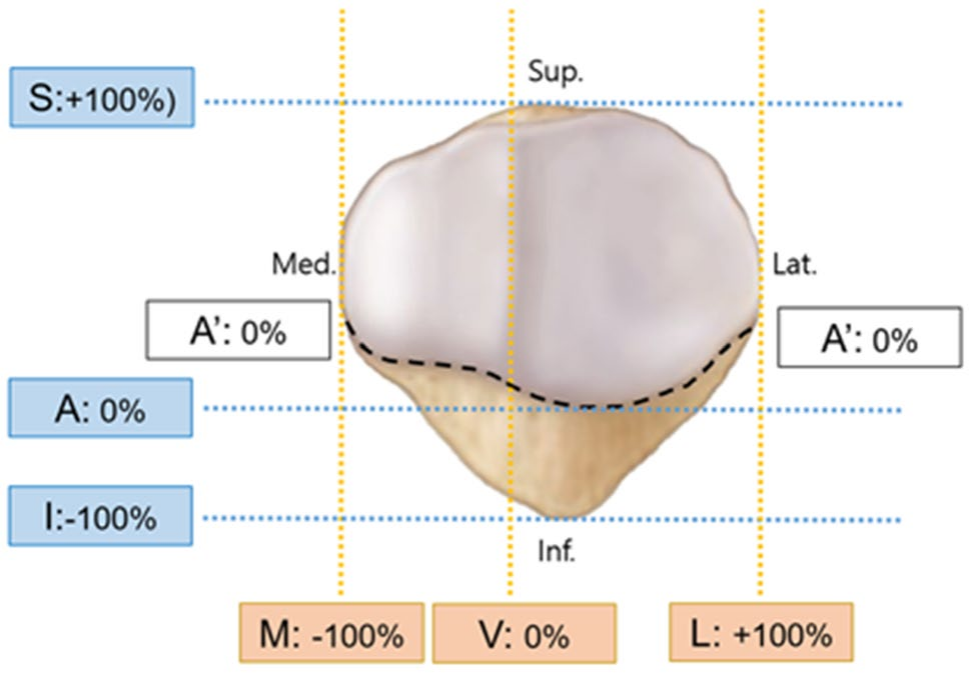
The reference lines for quantification of the fracture, Vertical references were S: the superior end of the patella (+ 100%), A: the lowest margin of articular facet (0%), I: inferior end of the patella (− 100%), A′: inferior boundary of the articular facet, Horizontal references were M: medial end of the patella (− 100%), V: vertical ridge, L: lateral end of the patella.

The fracture lines, classified as “primary horizontal-”, “primary vertical-”, “secondary horizontal-” and “secondary vertical-” fracture lines in the final denominated coronal articular fracture map, were digitized into coordinated values on the X and Y axes of a graph, and the lines were subtracted according to their classification. The mean value of each classified fracture line was calculated by averaging the coordinated value on the graph separately. To quantify the fracture lines in the coronal articular fracture map, the location of each fracture line was analyzed by the proportion to the separate reference line in the entire articular facet or extraarticular surface. The ‘primary horizontal’ fracture line was analyzed for how high the fracture line runs from the lowest margin of the articular facet at the separate horizontal points. The ‘secondary horizontal’ fracture line was analyzed how inferior the fracture line was located in the extraarticular surface from the inferior boundary of the articular facet. The ‘secondary vertical’ fracture line was analyzed how medial or lateral the fracture line was located from the medial and lateral margin of the patella (Fig. 2).

### Midsagittal fracture mapping

**Figure 3:**
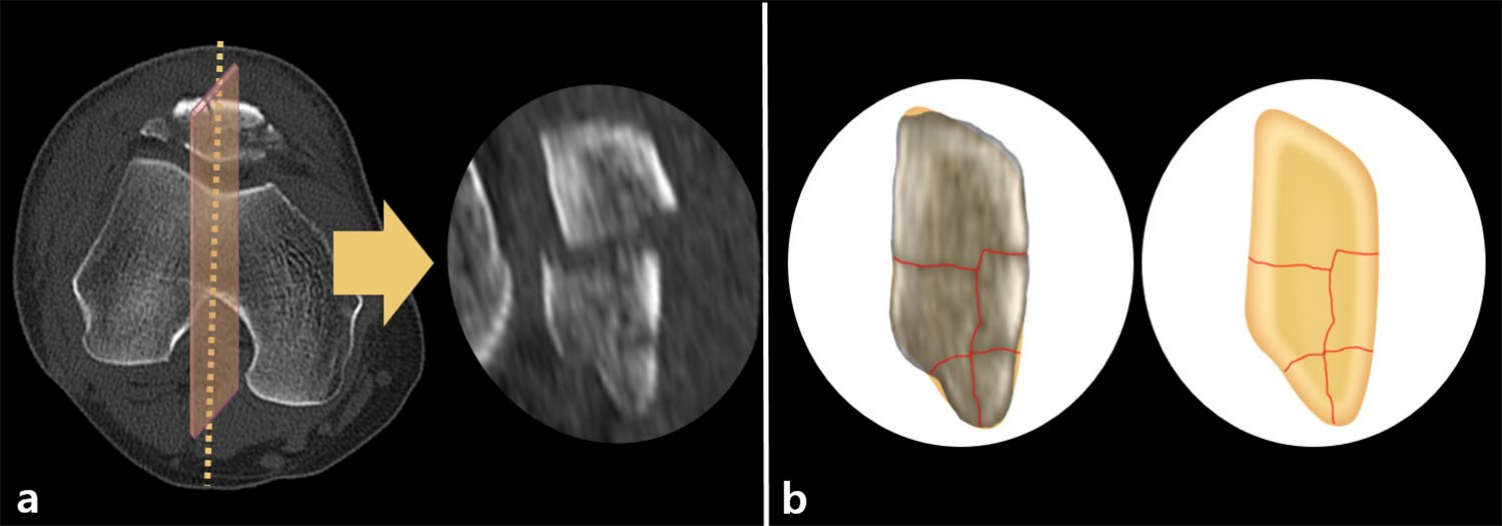
Two-dimensional computed tomography (CT) mapping of the midsagittal fracture pattern. A CT image of the patellar vertical ridge shows displaced and comminuted patellar fractures with coronal fragment and inferior pole fractures (**a**). The fracture fragments were reduced on the basis of a sagittal template. The fracture line (red line) was marked on the template (**b**).

We selected the sagittal DICOM images at the patellar vertical ridge to create a midsagittal fracture map. Based on our presumption of high incidence of coronal fragments at the boundary of the medial and lateral facets (without any selection bias), we selected the patellar vertical ridge as the reference because the vertical ridge can be easily identified by judging the axial scout images of CT scans. The identified fracture fragments on the midsagittal image were reduced by proper rotation and aligning along the anterior cortical and posterior articular margins. The fracture lines in each case were then individually reproduced on a standard patellar template of the midsagittal plane using Photoshop, similar to coronal articular fracture mapping (Fig. 3). After combining the data of the 66 fractures, the sagittal fracture map was superimposed onto a template.

### Mapping of the coronal split fragment zone

**Figure 4:**
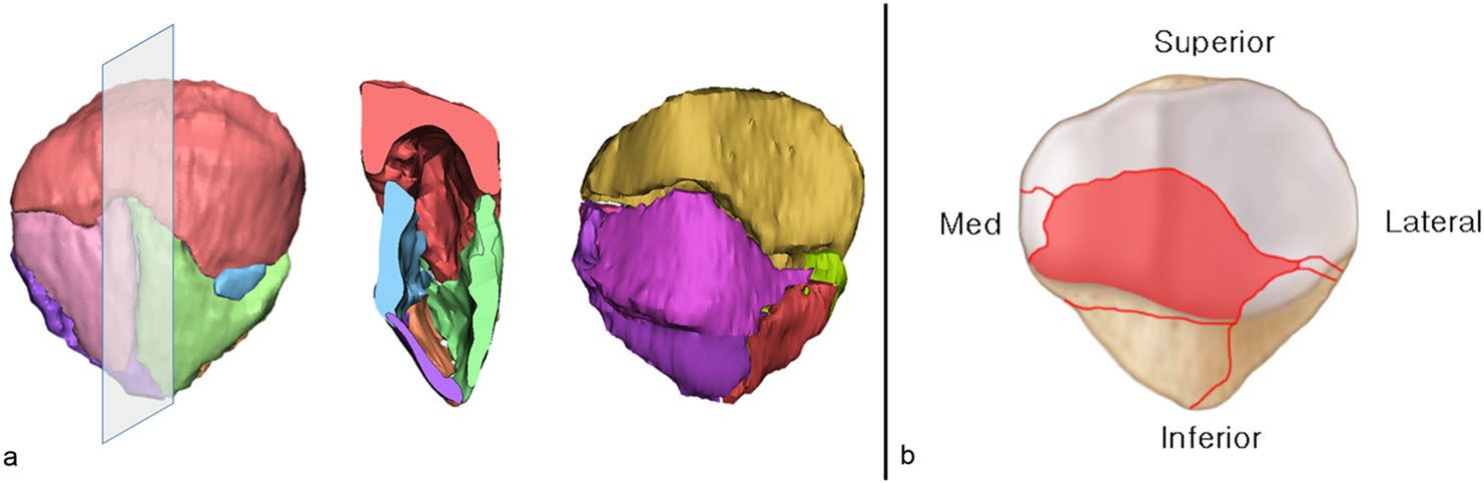
The method of mapping the coronal split fragment zone. In a case with articular step-off presented after the anterior cortex was perfectly reduced, the coronal split fragment was identified in a midsagittal cut and articular surface imaging (**a**). The zone of the coronal split fragment was marked on the coronal articular surface template (**b**).

Cases in which the articular surface step-off was > 2 mm, when the anterior cortex was perfectly reduced in 3D model, were suspected of having a coronal split fragment. By reviewing all CT images, we confirmed the presence of coronal split fragments and the configuration of the split (free articular or impacted type) (see Supplementary Fig. 1, which demonstrates the type of coronal split fragment). For mapping, these coronal fragments were segmented and reduced to achieve an anatomical reduction of the articular surface. Fragment locations were marked and painted on the template by the superimposition of articular surface images. After putting all the cases with coronal split fragments together in a single template, a combined mapping of the coronal split fragment zone was created (Fig. 4).

### Data analysis

The morphologic fracture map analysis was descriptive. For each categorical variable, we used Cohen’s kappa statistics to determine the interobserver and intraobserver reliabilities of the primary and secondary fracture lines. We also analyzed the factors possibly associated with the presence of specific fracture patterns (coronal split fracture or inferior pole fracture) using a logistic regression analysis. The statistical significance was set at a *P*-value < 0.05. All statistical analyses were performed using SPSS version 20.0 software (IBM, Armonk, NY, USA).

### Ethical approval

All experimental protocols were approved by Korea University Guro Hospital Institutional Review Board. All procedures and methods performed in studies involving human participants were in accordance with the ethical standards of the institution or practice at which the studies were conducted. (IRB number: 2019GR0364) Informed consent was obtained from all individual participants included in the study.

## Results

### Demographic characteristics

Among the 43 males and 23 females examined, the average age was 52 years (range 19–70 years) and the average body mass index (BMI) was 23.08 kg/m^2^ (range 15.8–34.6 kg/m^2^). The main injury mechanism observed among the patients observed was falling with the knee in a bent position (n = 49, 74.2%). “Tripping over while running” was the most common cause of injury. Associated injuries, besides patellar fractures, were noted in 18 patients. Detailed data, including age distribution, are described in Table 1.

**Table 1:** Patient demographic and fracture characteristics.

**Patients, n**	66
**Average age, years (range)**	51.7 (19–70)
**Age distribution, n (%)**	
19–29 years	2 (3.0%)
30–39 years	15 (22.7%)
40–49 years	10 (15.2%)
50–59 years	19 (28.8%)
60–70 years	20 (30.3%)
**Sex (M:F)**	43:23
**Laterality (R:L)**	32:34
**Body mass index, kg/m**^2^ (range)	23.1 (15.8–34.6)
**Open Fx. n, (%)**	5 (7.5%)
**Mode of injury, n (%)**	
*Fall with a bent knee position*	49 (74.2%)
Trip over while running	13
Trip over while walking	10
Fall on slippery floor	10
Tumble down the stairs	9
Fall while riding a bicycle	4
Vehicle–Pedestrian collision	3
*Fall from height of > 3 m*	8 (12.1%)
*Traffic accident*	9 (13.6%)
Dashboard injury in motor vehicle collision	5
Motorcycle collision	4
**Associated with injury besides patella, n (%)**	18 (27.2%)
Long bone fracture in the lower extremity	6
Multiple rib fractures	4
Facial bone fracture	3
Pelvic bone fracture	2
Brain concussion	1
Skull base fracture	1
Wrist fracture	1

*F* female, *Fx* fracture, *L* left, *M* male, *R* right.

### Coronal articular surface fracture mapping (Figs. 5 and 6)

**Figure 5:**
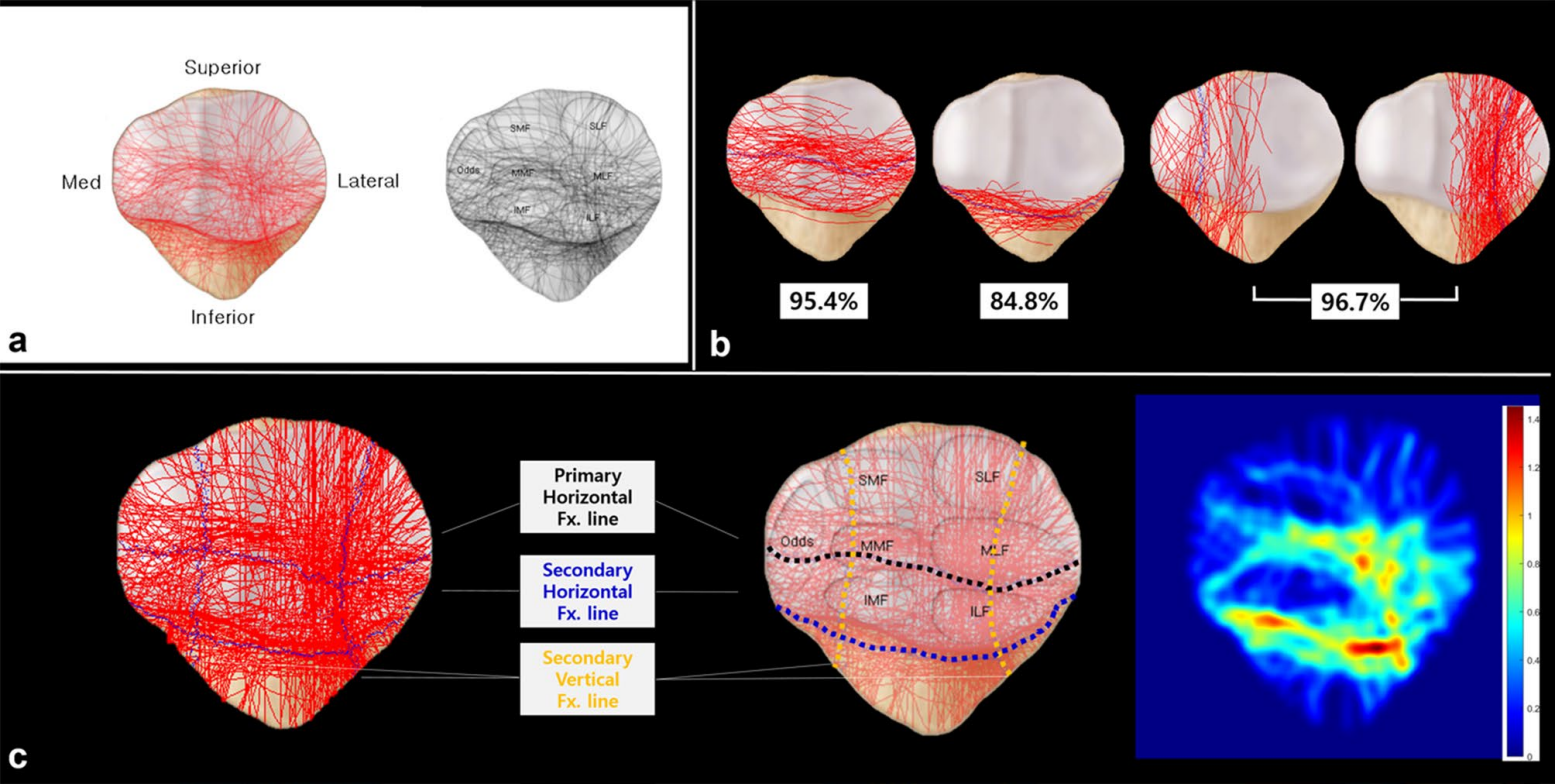
Coronal articular surface fracture mapping. This picture shows the fracture lines of all 66 superimposed fractures (**a**). The denominated fracture lines as a ‘Primary horizontal’, ‘Secondary horizontal’ and medial and lateral ‘Secondary vertical’ fracture line were digitized and the average value of each fracture line (blue dotted line) was calculated separately (**b**). Primary horizontal fracture lines (black dotted lines), secondary horizontal fracture lines (blue dotted lines), and medial and lateral secondary vertical fracture lines (yellow dotted lines) were deemed to be major fracture lines (**c**).

**Figure 6:**
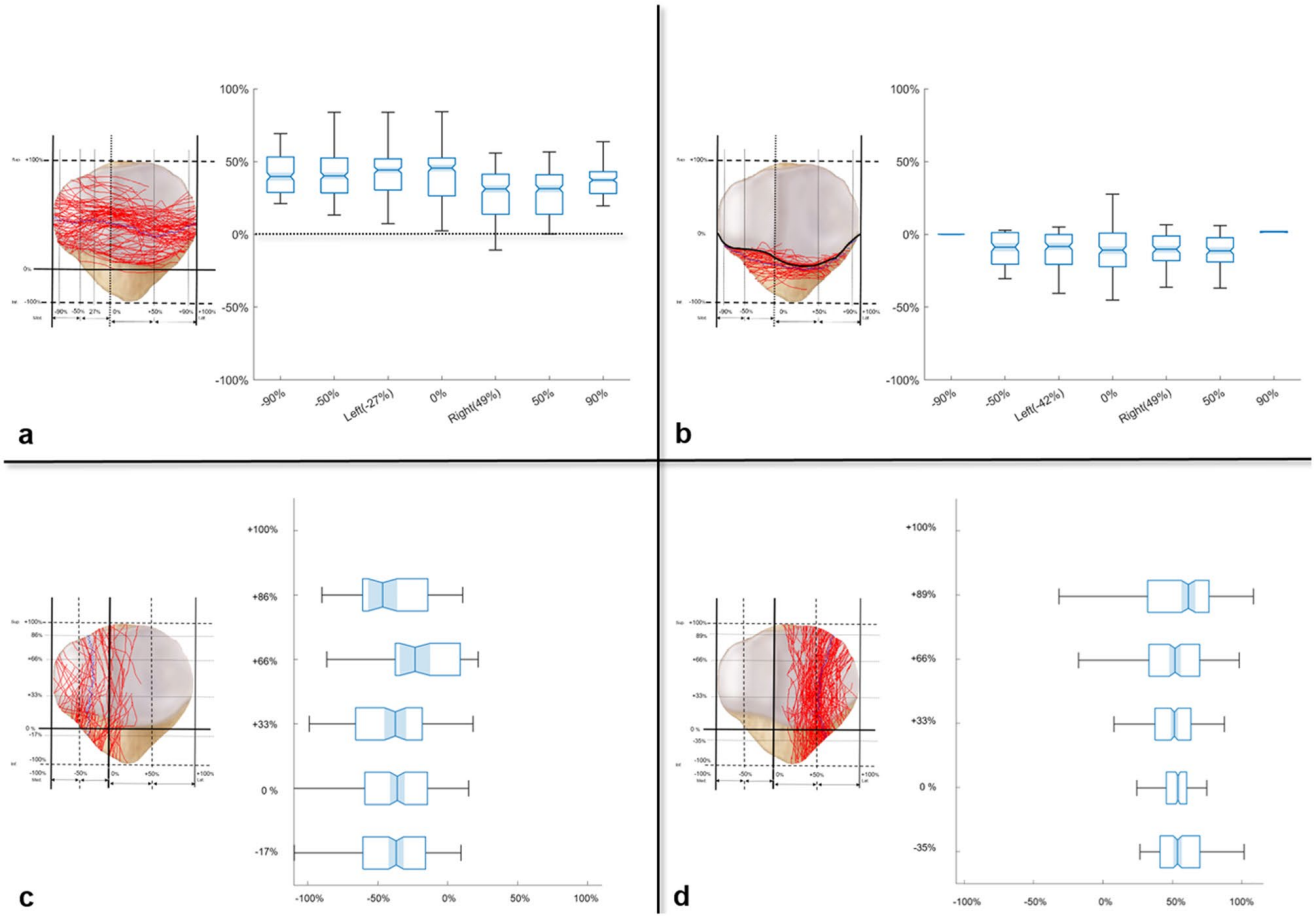
The distribution and the quantification of each fracture line, The average value of the primary horizontal fracture line runs ranged from + 27.9 to + 42.1% of the entire articular surface (**a**). The average value of the secondary horizontal fracture line was located within + 1.91 to − 12.6% from the inferior boundary of the articular facet (**b**). The average value of medial secondary vertical fracture line runs from − 52.2 to − 43.6% of a medial articular facet (**c**). The average value of lateral secondary vertical fracture line was located within + 27.4 to 44.6% of a lateral articular facet (**d**).

The primary fracture lines were classified into horizontal and vertical types. The primary horizontal fracture lines were identified in 63 patients (63/66, 95.4%). This line separated the proximal and distal fragments while breaking down the extensor mechanism. The average value of the primary horizontal fracture line was presented along the boundary between the middle and inferior facets on the articular surface. The level of the fracture line was measured as + 41.3% at − 90% point in medial facet, + 40.9% at the vertical ridge, + 28.3% at + 50% point in lateral facet, and + 36.4% at + 90% point in lateral facet. The primary vertical fracture lines were identified in only three patients.

The secondary fracture lines were also classified into horizontal and vertical types. The study identified secondary horizontal fracture lines in 56 patients (56/66, 84.8%). The average value of the secondary horizontal fracture line was run along the boundary of the articular facet and the inferior pole. From the inferior boundary of the articular surface, the secondary fracture line's level ranged from + 1.91 to − 12.6%. The inferior pole fracture, which is extra-articularly avulsed by the patellar tendon, was induced by a secondary horizontal fracture line and observed in 55 cases (55/66, 83.3%). Comminution of the inferior pole fracture was also observed in 30 cases (30/55, 54.5%). Secondary vertical fracture lines were identified in 64 patients (64/66, 96.7%). Located at the periphery of lateral or medial facets, including the odd facet, these fracture lines were connected to the primary fracture line and produced satellite fragments. Satellite fragments were found on medial odd facets in eight patients (8/64, 12.5%), on lateral facets in 27 (42.2%), and on both, medial odd and lateral, facets in 29 (45.3%).

The interobserver agreement of the classification of fracture lines had a Cohen’s kappa value of 0.787 (95% confidence interval [CI] 0.691–0.874) for the primary and 0.725 (CI 0.622–0.841) for the secondary fracture lines. Intraobserver agreement, performed by the first observer, resulted in a Cohen’s kappa value of 0.822 (CI 0.746–0.932). There was substantial agreement with interobserver reliability and strong agreement with intraobserver reliability.

### Midsagittal fracture mapping

**Figure 7:**
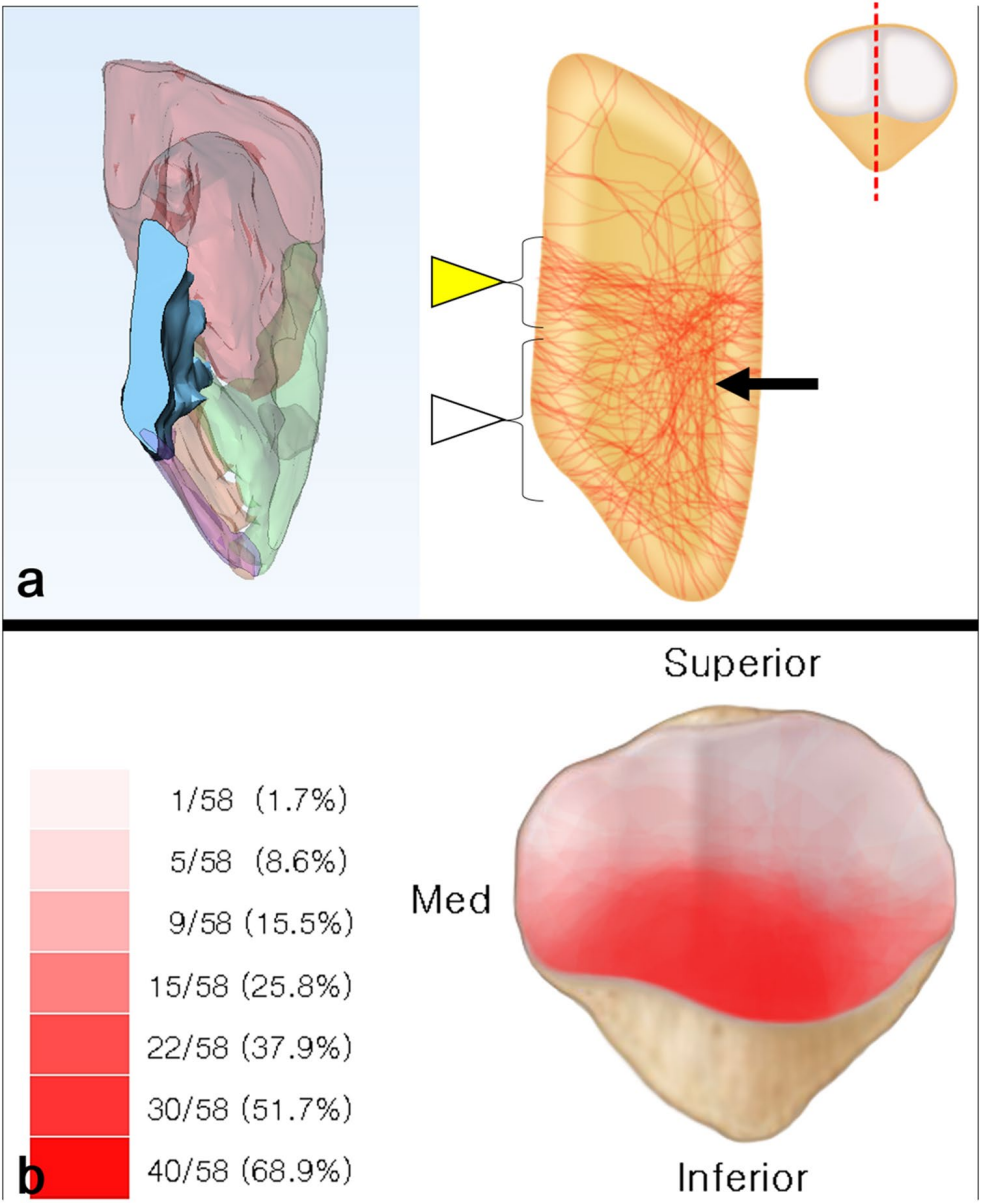
Midsagittal fracture mapping showing an X-shaped fracture pattern made of primary horizontal fracture lines (yellow arrow), secondary fracture lines (white arrow), and main coronal fracture lines (black arrow). By linking these fracture lines, the coronal split fragment can be developed (**a**). This picture shows the distribution of the coronal split fragment. The major zone of the coronal split fragment was the inferior facet of the lateral and medial facets (**b**).

The study identified an X-shaped fracture line representing primary and secondary horizontal fracture lines. Between the primary and secondary horizontal fracture lines, a fracture connection called the “main coronal fracture line” exists. This fracture line is mostly located at the anterior third of the midsagittal view. The development of coronal split fragments can be identified in the combination of primary horizontal, secondary horizontal, and main coronal fracture lines (Fig. 7a).

### Mapping of coronal split fragment zone

The coronal split fragment was observed in 58 cases (87.9%). The most common area of the coronal split fragment was the inferior facet of the lateral and medial facets. The fragments were of the free articular type in 32 cases (55.2%) and the impacted type in 26 cases (44.8%) (Fig. 7b).

### Factors affecting specific fracture patterns

Age, gender, BMI, injury mechanism, and the presence of primary horizontal and secondary vertical fracture lines were not statistically significant concerning the occurrence of inferior pole or coronal split fractures. The presence of the free articular type of coronal split fragment was significantly related to inferior pole fractures (odds ratio, 8.15; 95% confidence interval [CI] 2.39–27.6; *P* = 0.01).

## Discussion

The fracture mapping technique described by Cole et al.^14^ was applied in this study to evaluate the fracture patterns of AO/OTA 34 C3 multifragmentary patellar fractures. To the best of our knowledge, this technique has been used to identify the fracture patterns of complex articular fractures, including scapular fractures^15^, pilon fractures^14^, tibial plateau fractures^16,17^, posterior malleolar ankle fractures^18^, Hoffa fractures^19^, distal radius fractures^20^, and quadrilateral plate fractures^21^. However, this is the first study to analyze the articular fracture patterns systemically in the coronal and sagittal views to improve the understanding of complex articular injuries of the patella.

The traditional classification system of patellar fractures is based on plain radiographs and articular fracture patterns are presented in the frontal view of the patella^22,23^. Thus, a limitation of the classification system is characterization of the articular fracture patterns and comminution^5^. In the recent AO/OTA classification, the fracture patterns were described in the coronal, sagittal and axial planes, classifying complex articular fracture patterns as complete articular frontal/coronal simple (C1), wedge (C2), and multifragmentary (C3) fractures. However, there is no further subgroup categorization in the multifragmentary group describing detailed fracture patterns in the multiaxial plane^13^. Recently, Lazaro et al.^8^ highlighted the clinical impact of CT scans delineating the fracture patterns of patellar fractures and demonstrated the necessity of CT-based classification accounting for the associated inferior pole fractures. We also believe that actual fracture patterns and comminution in the coronal articular and the sagittal planes have not been reflected in the current classification. Thus, this system may not be suitable in the clinical setting for understanding the complex articular injuries of the patella, besides the establishment of the sequence of fracture reduction and selection of optimal fixation methods in complex patellar fracture management. Therefore, our findings may be used to provide substantial information for the development of new AO/OTA classification systems for patellar fractures.

In this study, we created coronal articular and midsagittal fracture maps from images of CT scans of the 66 multifragmentary (AO/OTA 34C3) patellar fractures in our cohort to analyze articular fracture patterns systemically in the coronal and sagittal planes. Our descriptive analysis of the coronal articular fracture map revealed that multifragmentary patellar fractures tend to have three fracture lines: a primary horizontal fracture line that compromises the largest displacement at the middle and inferior facet levels of the patellar body; a secondary horizontal fracture line at the inferior margin of the articular facet; and a secondary vertical fracture lines which compromise satellite fragments on medial odd facet, on lateral facet or on both. Furthermore, the midsagittal fracture map identified that these fracture lines result in a combined inferior pole fracture and coronal split fragment at the inferior facet of the lateral and medial facets, with the collaboration of the main coronal fracture line.

There are several clinical implications of improved understanding of multifragmentary patellar fracture morphology, including the morphology described in this study, with respect to facilitating successful treatment of multifragmentary patellar fractures (see Supplementary Fig. 2, which demonstrates the clinical case). First, the presence of a primary horizontal fracture line that runs through the middle or inferior facet can be used as a guideline for main fracture fixation. Although many surgical options for stabilizing multifragmentary patellar fractures have been introduced, there remains no consensus regarding fracture fixation^24,25^. The presence of primary horizontal fracture lines can require the use of tension-band construct fixation even in multifragmentary fracture patterns^1,26^. However, comminuted articular fractures need to be reconstructed anatomically before fixing the main fractures to ensure fracture site compression in tension-band construct fixation^27^. Tension-band fixation augmented with other supplementary fixatives, including mini plates or screws, multiplanar tension-band wiring, and plating on the tension side, are good examples for which this rationale is used^9,28^.

The second implication of our study relates to the presence of coronal split fracture fragments. Most cases (up to 87.8%) had coronal split fragments of either the free articular or the impacted type. These findings highlight the difficulties and risks involved in articular reduction. The most widely used surgical approach is the anterior approach, in which articular reduction is judged by reducing the anterior cortex indirectly, either by intraoperative C-arm imaging or by palpating articular reduction through the retinacular tear^26^. If coronal split fractures are not managed before main fracture reduction, there is a high risk of articular malreduction. There are many surgical strategies for managing coronal split fragments, including changing the surgical approach. An alternative surgical approach is the lateral parapatellar approach. Gardner et al.^29^ suggested using the lateral parapatellar approach and direct reduction to manage comminuted articular fractures. By everting the patellar articular surface, direct visualization for the treatment of complex articular fractures is achieved. An additional method involves reducing the coronal fragment through the fracture window. The coronal split fragment is created by primary and secondary fracture lines. Through the fracture window made from the main primary horizontal fracture line, reduction and fixation of the coronal fragments can be achieved. Moreover, these coronal split fragments can be classified into two types: free articular and impacted. According to the coronal split fragment type, reduction and fixation can differ. If fragments are of the free articular type, they can be reduced back to either proximal or distal main fragments through the fracture window and fixed with imbedded mini-screws or through anterior cortical plating. If fragments are of the impacted type, the fracture can be dis-impacted through the fracture window and the defect can be filled with allograft bone chip grafts^9^.

The third implication is the involvement of inferior pole fractures. Similarly with the rate of comminuted inferior pole fracture in the previous report^8^, the results obtained in our study demonstrate that most cases (83.3%) were associated with inferior pole fractures. Moreover, more than half of them (54.5%) showed comminution with a sagittal split. This finding suggests that caution must be taken to manage associated inferior pole fractures in order to prevent unexpected breakdown of the extensor mechanism during the active rehabilitation phase. Several supplementary surgical options for managing associated inferior pole fractures and their coverings have to be planned preoperatively; these include adding cerclage wiring, suturing distal pole fragments, rim plate augmentation, basket plating, and multiplanar plating^10,29–34^. Furthermore, these findings have to be reflected in the design for precontoured plates in patellar fractures^34^.

In our study, the free articular type of coronal split fragment was statistically associated with the presence of an inferior pole fracture. This finding can be explained in the midsagittal fracture map as the coronal fragment, which is mostly located on the inferior facet and has a distal fracture split exiting on the articular side as a free articular fragment. This split line is associated and connected with the secondary horizontal fracture line, resulting in an inferior pole fracture. This finding suggests that an associated injury can be predicted based on fracture morphology. Therefore, if free articular coronal fragments are observed on plain radiographs, the associated inferior pole fractures should be carefully examined using CT scans, when surgical treatment is performed^8^.

There are several limitations of this study. First, the possibility of selection bias cannot be disregarded. Most injuries in our cohort resulted from falling down on a bent knee. Patients sustained multifragmentary patellar fractures by tripping over while running, tumbling down the stairs, and through other moderate energy causes of injury involving a bent knee position. However, enrolling a comparable number of high-energy injuries associated with direct hit, such as dashboard injuries in motor vehicle collision, would be complicated. Thus, our results are applicable only for cases involving low- to moderate-energy trauma. Second, the reduction of fractures in the model using 3D software was manually performed. In current 3D software systems, no auto-reduction function exists in the fracture model. To limit the risk of technical errors, all procedures were performed by qualified technicians, and each process was supervised and confirmed by two senior surgeons. Third, our analysis of fracture patterns lacks the clinical relevance of proven clinical data. There is still some doubt regarding how this fracture mapping technique can have notable clinical impact. Prospective cohort studies comparing the clinical results between groups treated based on 3D CT fracture pattern analysis and control groups treated based on conventional classification should be conducted in the future. We believe that articular fracture mapping along the articular surface, rather than frontal imaging of figures as in the conventional classification system, can enhance understanding of the articular fracture pattern configuration and systemize the stabilization strategy, with an emphasis on articular reduction.

To our knowledge, this is the first study to elucidate common articular fracture lines and associated fracture patterns, including coronal split fragment and inferior pole fractures, using a 3D fracture mapping technique for AO/OTA 34 C3 multifragmentary patellar fractures.

## Conclusion

Mapping multifragmentary patellar fractures revealed that comminuted patella fractures have a primary articular horizontal fracture line with secondary vertical and horizontal fracture lines, resulting in coronal split fragments (87.8%) and inferior pole fractures (83.3%).

## Supplementary Information

Supplementary Legends.

Supplementary Video 1.

Supplementary Figure 1.

Supplementary Figure 2.
