# Supplementary material for: Multifragmentary patellar fracture has a distinct fracture pattern which makes coronal split, inferior pole, or satellite fragments
Source: Sci Rep. 2021 Nov 24;11:22836. doi: 10.1038/s41598-021-02215-0 (PMC8613236; doi:10.1038/s41598-021-02215-0)
Supplement: Supplementary file 1 — Supplementary Legends. [file 41598_2021_2215_MOESM1_ESM.docx]

Supplementary figure 1, Type of coronal split fragment, the free articular type was diagnosed when the coronal fragment was completely separated with a loss of bone continuity from the proximal or distal axial fragments requiring coronal, sagittal, and axial reduction of the fragment. (yellow arrow head represents the exit of fracture line of coronal split fragment) (a). The impacted type was diagnosed when the fragment only had one plane deformity with an intact distal subchondral cortical hinge (white arrow head) (blue bi-arrowed line represent the width of proximal fragment, red bi-arrowed line represent the width of distal fragment, the dotted white lines represent a step off between two lines) (b)

Supplementary figure 2, A 69 year old male sustained a multifragmentary patellar fracture of the Rt. knee (a), Coronal articular fracture mapping shows the Primary horizontal fracture line (black dotted line) is separating the proximal and distal main fragments, Secondary horizontal fracture line (blue dotted line) creates an inferior pole fracture fragment (yellow oval symbol) and Secondary vertical fracture line (yellow dotted line) creates satellites fragments (pink heart symbol). The combination of these fracture lines results in a coronal split fracture fragment (blue star symbol) (b), Coronal split fragment was fixed with two embedded 1.5mm screws through the primary horizontal fracture window. Upper satellite fragment and proximal main fragment was fixed with anterior cortical plating. Primary and Secondary horizontal fracture lines were reduced with a pointed reduction clamp. Tension band plating was applied over the inferior pole fracture fragment (c), At 3 months follow up, fracture was united and range of motion was recovered fully (d).
