# Supplementary figures and images for: Multifragmentary patellar fracture has a distinct fracture pattern which makes coronal split, inferior pole, or satellite fragments
Source: Sci Rep. 2021 Nov 24;11:22836. doi: 10.1038/s41598-021-02215-0 (PMC8613236; doi:10.1038/s41598-021-02215-0)

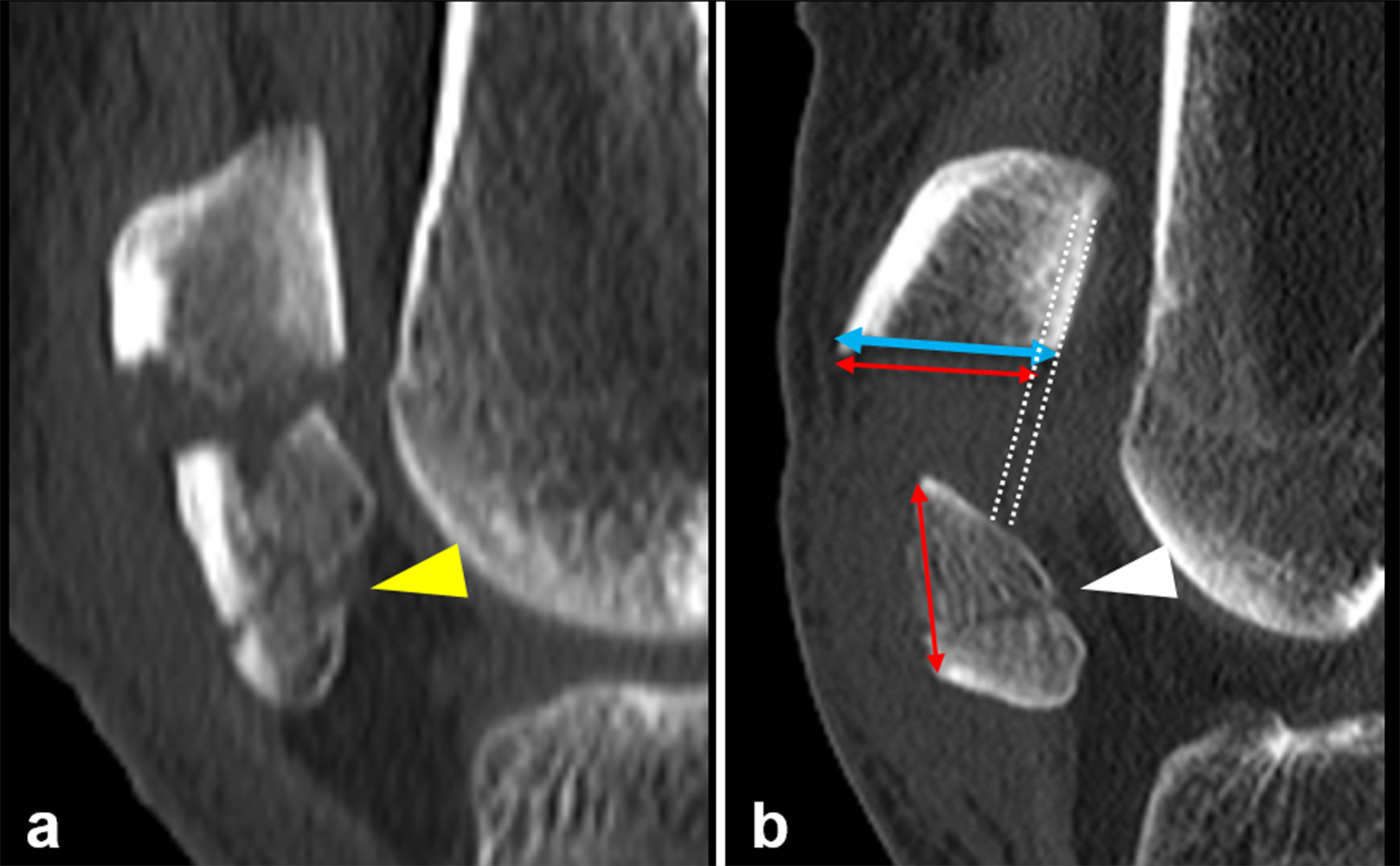

Supplement: Supplementary file 3 — Supplementary Figure 1. [file 41598_2021_2215_MOESM3_ESM.tif]

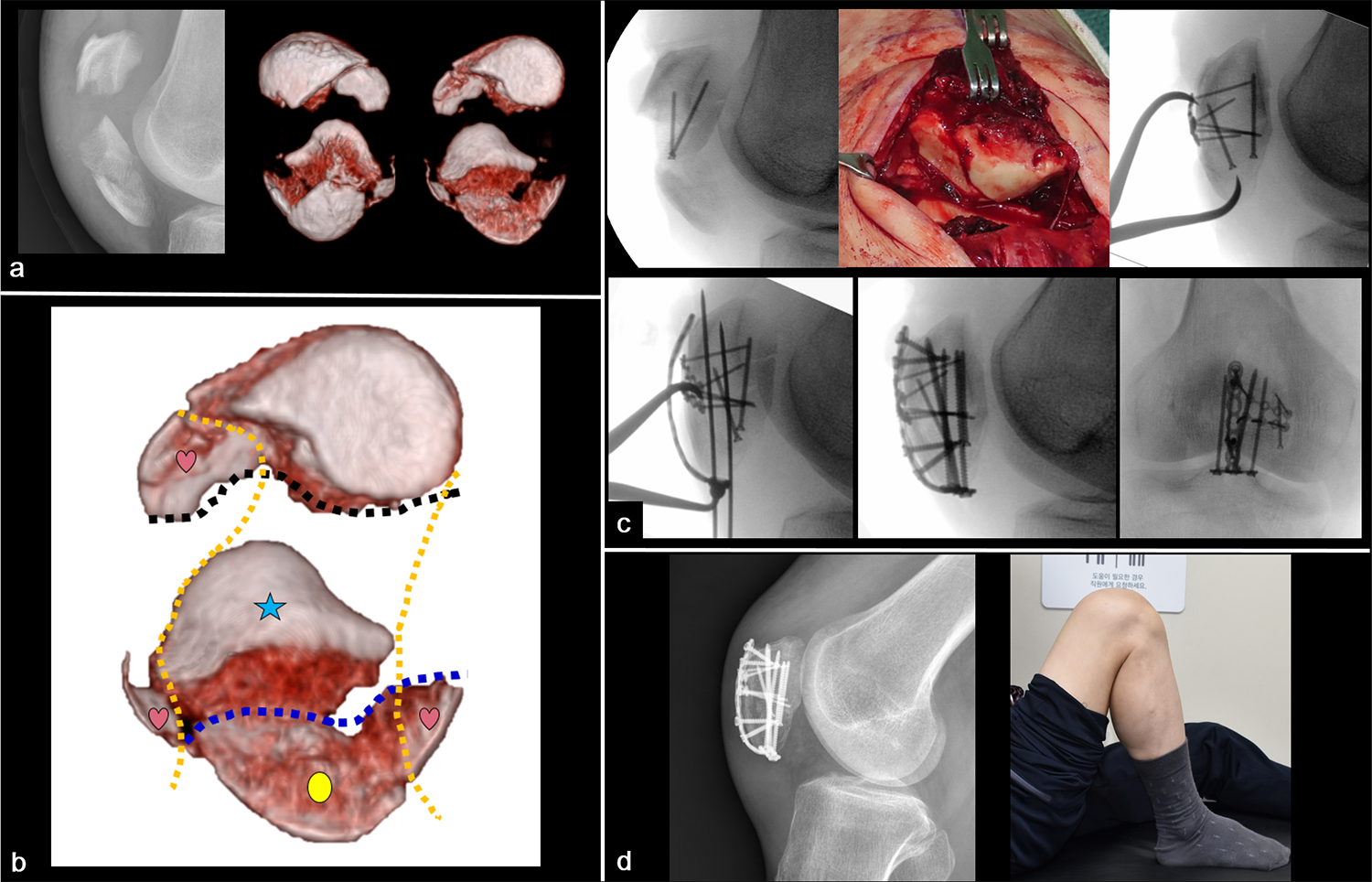

Supplement: Supplementary file 4 — Supplementary Figure 2. [file 41598_2021_2215_MOESM4_ESM.tif]
